# The simultaneous presence and expression of human hepatitis C virus (HCV), human herpesvirus-6 (HHV-6), and human immunodeficiency virus-1 (HIV-1) in a single human T-cell

**DOI:** 10.1186/1743-422X-4-106

**Published:** 2007-10-24

**Authors:** S Zaki Salahuddin, Katherine A Snyder, Andre Godwin, Renu Grewal, John G Prichard, Ann S Kelley, Dennis Revie

**Affiliations:** 1 Department of Basic Research, California Institute of Molecular Medicine, Ventura, California, USA; 2 Department of Biology, California Lutheran University, Thousand Oaks, California, USA; 3 Departments of Medicine and Family PracticeVentura County Medical Center, Ventura, CA; 4 Department of Hematology and Oncology, Ventura County Hematology-Oncology Specialists, Ventura, CA

## Abstract

We have developed a system that isolates and replicates HCV *in vitro*. These isolates are called CIMM-HCV. This system has made it possible to analyze the biology, nature, and extent of HCV variability, among other things. Individuals that are infected with HIV-1 are often also infected with HCV and HHV-6. In addition to HCV, our lab has systems for replicating HIV-1 and HHV-6. We asked whether all these viruses could infect the same cells. We report here the successful infection of a T-cell (CEM) by CIMM-HCV, HHV-6, and HIV-1. PCR analyses demonstrated that the CEM cells were productively infected by HHV-6A. RT-PCR showed that the same cell culture was positive for HCV and HIV-1. Co-infection of a T-cell by all three viruses was confirmed by transmission electron microscopy (TEM). All these viruses are highly cytolytic; therefore, triply-infected cells were short lived. However, HIV-1 and HCV co-infected cells unexpectedly lasted for several weeks. Viral replication was unhindered and the phenomenon of 'dominance' was not observed in our experiments. In addition, CIMM-HCV was present in the perinuclear space, suggesting their possible synthesis in the nucleus. This report is based entirely on viruses produced *in vitro *in our laboratories. As part of the determinations of host ranges of these viruses, studies were designed to demonstrate the infection of a single cell by these viruses and to study the consequences of this phenomenon. All measurements were made on cultured cells and cell culture supernatants.

## Background

Individuals harbouring more than one virus in acute or chronic diseases are frequently observed. A minority of patients that are infected with HIV-1 are at least doubly infected [1]. Infection with HIV-1, HCV, and human hepatitis B virus (HBV) may result from a common route of infection. The majority of these individuals are also infected with HHV-6, and other DNA viruses such as Epstein-Barr virus (EBV) or Cytomegalovirus (CMV). Except for HIV-1, many of these viruses co-exist in healthy individuals without causing any pathological consequences. The host range of all these viruses is well known.

Although co-infection by HIV-1 and HCV has been extensively studied in AIDS patients, *in vitro *studies of co-infected cell cultures are few and limited in scope. AIDS patients are frequently infected with HHV-6 in addition to HIV-1 and HCV. In general, HIV-1 adversely affects HCV-infected patients while the effects of HCV on HIV-infected patients are less defined and controversial [2,3]. Increased morbidity in co-infected individuals would not be surprising. HAART, however, may have changed the dynamics of AIDS by prolonging the lives of HIV-infected individuals irrespective of infections and other associated problems [4].

Our effort is to understand the impact of multiple infections at a cellular level, both on virus reproduction and its characteristics and on their effects on cell functionality. This may help explain some pathogenic consequences such as neuropathy or dementia.

## Results

We previously reported an *in vitro *system that can replicate HCV for extended periods of time [5]. Later reports from our laboratories included an analysis of the 5'UTR of CIMM-HCV [6] and the discovery of significant HCV variants [7]. Rare insertions and deletions have also been seen by others [8]. The analysis of the 5'UTR revealed that there were no significant differences between HCV-RNA found in patient's blood and the CIMM-HCV.

### Host ranges of each virus

Since HIV-1, HCV, and HHV-6A are routinely isolated in our laboratories, it was decided that we should determine whether these agents can co-infect the same cells. In developing our co-infection system, we first needed to select a cell line that could be infected by all three viruses (Table 1). Macrophages and T-cells were the most suitable cell types for our co-infection experiments. B-cells do not lend themselves to studying this group of viruses, as HIV-1 and HHV-6 are T-cell tropic agents. Since T-cells are easier to continuously culture than macrophages, and also more productive, CEM were selected as target cells.

**Table 1:** Summary of viral transmission experiments with various hematopoetic and liver cells

	**HCV short term**	**HCV long term**	**HIV**	**HHV-6**
**A. T-cells**^1^	+	-	+	+
**B. B-cells**^2^	+	+	-	-
**C. Monocytes/macrophages**^3^	+	-	+	+
**D. Neuronal precursors**^4^	+	+	+	+
**E. Liver cells**^5^				
**Kupffer's**	+	-	+	+/-
**Hepatocytes**	+	+/-	-	-

^1^T-cells isolated from human fetal chord blood.

^2^B-cells immortalized by infection with transforming EBV.

^3^Monocyte/Macrophages, adherent cells stimulated with PMA.

^4^Recently isolated neuronal cells from fetal human brain.

^5^Freshly isolated liver cells from liver biopsies. Kupffer's cells are liver macrophages and Hepatocytes are liver endothelial cells.

### Infection of T-cells by all three viruses

**Figure 1:**
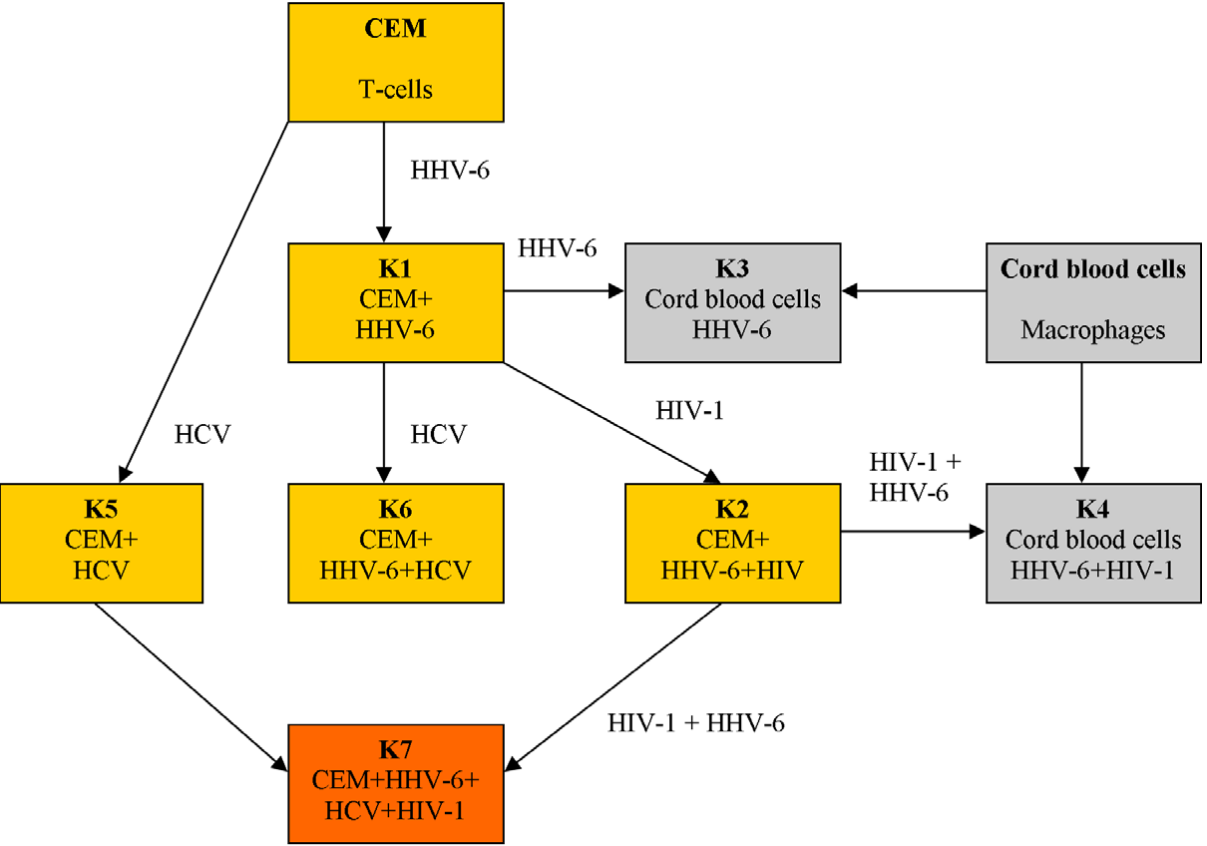
**Schematic showing the infection process**. The viral transmissions used cell-free supernatants. The designations K1 through K7 don't represent the order of the experiments. The respective viruses and cell types for each cell culture are indicated in the boxes.

**Figure 2:**
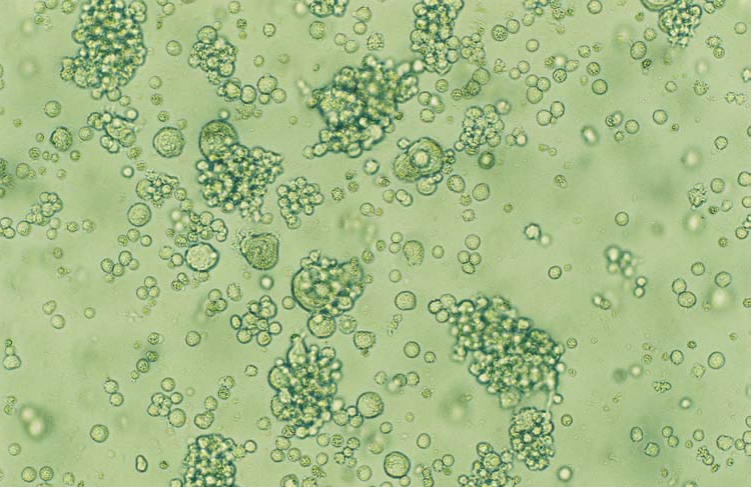
**Cytopathic effects in CEM cells**. This picture represents the commonly observed cytopathic effects in triply-infected CEM cells. Large cells are seen with increasing frequency in infected cultures.

**Figure 3:**
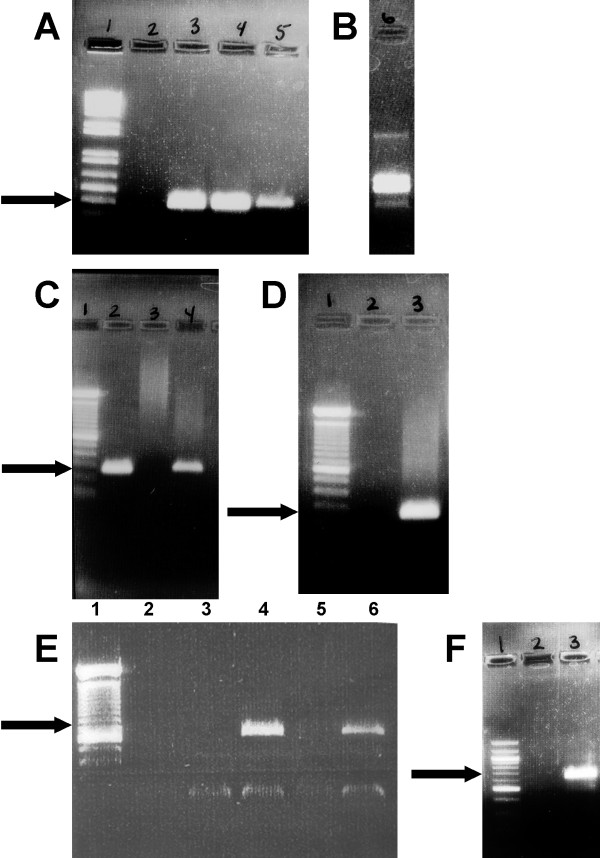
**Agarose gels of PCR products.** The DNA standard is a 1 kb plus ladder (Invitrogen). Arrows indicate the expected band sizes. Positive bands for K7 are seen in gel B lane 6 (HHV-6), gel C lane 4 (HCV), and gel F lane 3 (HIV-1). **A.** HHV-6. Lane 1 is the ladder, Lane 2 is uninfected CEM cells, Lane 3 is K1, Lane 4 is K2, and Lane 5 is K3. The positive bands are 401 bp in size. **B.** HHV-6. Lane 6 is K7. This sample was run on a different gel than gel A. **C.** HCV. Lane 1 is the ladder. Lane 2 is K5, Lane 4 is K7. The positive bands are 269 base pairs in size. **D.** HCV. Lane 1 is the ladder. Lane 3 is K6. **E.** HIV-1. Lane 1 is the ladder. Lane 2 is uninfected CEM cell supernatant, Lane 3 is K1, Lane 4 is K2, Lane 5 is K3, and Lane 6 is K4. The positive bands are 650 base pairs in size. **F.** HIV-1. Lane 1 is a 100 bp ladder (Invitrogen). Lane 2 is K6 and Lane 3 is K7.

CEM cells were infected individually by each of the three viruses (Fig. 1). To test the infectivity of HHV-6A, CEM cells were infected with the virus (Figure 1, K1) [9,10]. Two methods were used to determine whether the cell culture was infected and actively producing HHV-6A particles. First, characteristic cytopathic effects (CPE) on the cells were observed. The cells have the appearance of a balloon, called "juicy cells" (Figure 2) [10]. Second, the cell culture supernatants were tested by PCR using the appropriate primer set (Table 2). A band of about 400 bp was seen after PCR analysis, indicating HHV-6A was replicating in these cells (Figure 3A, Lane 3).

**Table 2:** Primers used to analyze HHV-6A, HCV, and HIV

**Virus**	**Primer**	**Sequence (5' to 3')**	**Reference**
HCV	HCV 9.1	gac act cca cca tag atc act c	[5]
HCV	HCV 9.2	cat gat gca cgc tct acg aga c	[5]
HCV	HCV 10.1	ctg tga gga act act gtc ttc acg cag	[5]
HCV	HCV 10.2	cac tcg caa cca ccc tat cag	[5]
HIV	Con-1f1	cca gcn cac aaa ggn ata gga gg	[33]
HIV	Con-1r1	acb acy gcn cct tch cct ttc	[33]
HIV	Con-1r2	ccc aat ccc ccc ttt tct tta aaa tt	[33]
HHV-6	U16-17F	cgt aga aca gaa gac cgg c	[34]
HHV-6	U16-17R	aga act gca aat cgt tcc g	[34]

CEM cells were infected separately with HCV (K5). Evidence of HCV was seen after RT-PCR (Figure 3C, Lane 2). CEM cells were also separately infected with HIV-1 (data not shown). All three viruses could productively infect CEM cells.

The general procedure we used to produce triply-infected cells was to sequentially infect CEM cells with the viruses. For a successful co-infection with multiple viruses, the order of infection of a cell type may determine whether the experiment will succeed. In our case, to produce the triply-infected cells, infection proceeded with CIMM-HCV, followed by HHV-6A and HIV-1 (Figure 1). Cell cultures were incubated overnight post infection and allowed to incubate at 37°C for the remainder of the experiment.

HHV-6A infected CEM cells (K1) were infected with HCV using our standard infection process to produce K6. The presence of HCV was determined by RT-PCR (Figure 3D, lane 3). Our results demonstrate that the CEM cells were co-infected with both HHV-6A and HCV (Figure 1, K6).

HHV-6A infected CEM cells were also infected separately with HIV-1 (Figure 1, K2). The presence of HIV-1 was determined by RT-PCR (Figure 3E, Lane 4). We therefore demonstrated that CEM cultures could be co-infected by the combinations of two different viruses (cultures K2 and K6).

As a control, we infected macrophages obtained from cord blood cells with HHV-6A (K3) or both HHV-6A and HIV-1 (K4). We were able to show infection of these cells by each virus (Figure 3A, Lane 5; Figure 3E, Lanes 5 and 6).

Finally the HCV infected K5 cells were infected with HHV-6A and HIV-1 from culture K2. The presence of HCV (Figure 3C, lane 4) and HIV-1 (Figure 3F, Lane 3) were determined using RT-PCR, and HHV-6A by PCR (Figure 3B, Lane 6). In short, the CEM cells were found to be simultaneously infected with all three viruses (Figure 1, K7).

The triply infected CEM cells were observed by light microscopy for CPE. The cells looked polymorphic compared to uninfected cells. This has been reported previously for HHV-6A [10], and HIV-1 [11], and HCV [5]. All three viruses are cytolytic, so triply infected CEM cultures lasted for approximately three weeks. The HHV-6A infected cells were the first to die. They were followed by HIV-1 infected cells and finally the HCV infected ones died. At this point these experiments were terminated.

### Infection of single cells

Since PCR analysis showed that all three viruses were present in the same cell culture, the next question was whether individual cells were similarly infected. To answer this question, we prepared samples for transmission electron microscopy (TEM).

**Figure 4:**
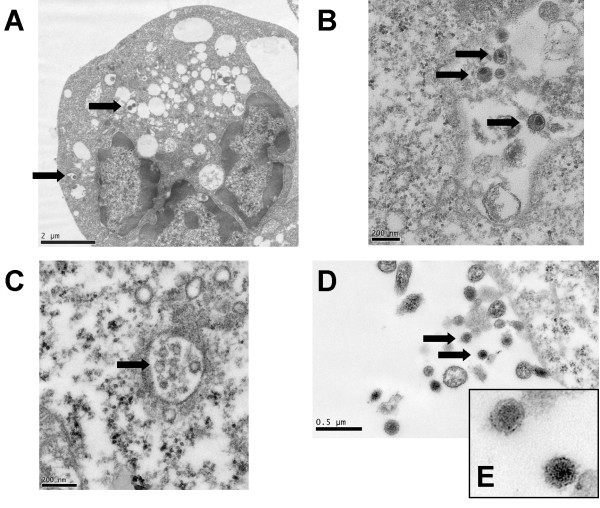
**Electron micrographs of infected cells**. **A**. K6 cell infected with HHV-6 (arrows) with margination of the chromatin in the nucleus and extensive vascularization. **B. **K2 cell showing HIV-1 particles (arrows). It is unclear whether the particles are outside the cell or in a vacuole inside the cell. **C. **K5 cell showing partial HCV particles inside a vacuole (arrow). **D. **K6 cell showing HCV particles (arrows). **E. **Inset showing HCV from Figure 4D.

Due to its large size and distinct morphology, TEM pictures of cells infected only with HHV-6A (K1) were easily identified (Figure 4A). The intracellular particles were around 150 to 200 nm in size. This allowed us to compare the HHV-6A infected cells with cells co-infected with HHV-6A and HIV-1 (K2). Distinct maturation of these viruses made it easy to determine their presence (Figure 4B).

TEM pictures of cells infected with HCV (K5) were examined next. Complete as well as incomplete HCV particles were observed in the cytoplasm of infected cells (Figure 4C and 4D). The complete HCV particles were 70 to 100 nm in size, while the incomplete particles were 50 to 70 nm in size. The HCV virions and the incomplete HCV particles resembled pictures published by other investigators [12,13].

CEM cells that were co-infected with HHV-6A and HCV (K6) were also examined. The presence of both HHV-6A and HCV, including immature HCV particles inside the vesicles were noted.

TEM pictures show that HIV-1 and HCV were generally of similar size. In our analysis, HIV-1 usually appeared to be a little larger than HCV, which may be an artefact of fixation and processing.

**Figure 5:**
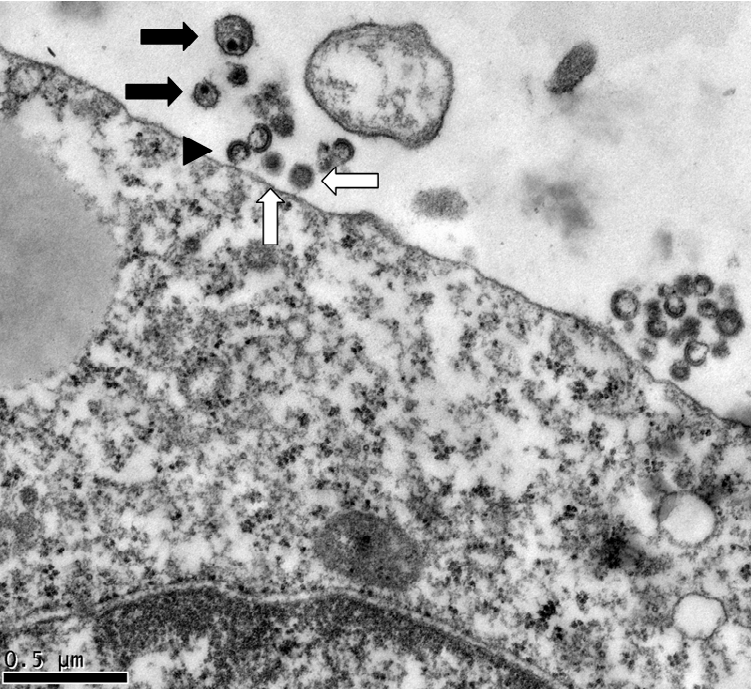
**Electron micrograph of HCV and HIV-1 extracellular particles in the vicinity of a K7 cell**. Representative HIV-1 particles are indicated with black arrows, HCV with white arrows, and immature HIV-1 particles by an arrowhead. The HIV-1 particles are a little larger than HCV.

**Figure 6:**
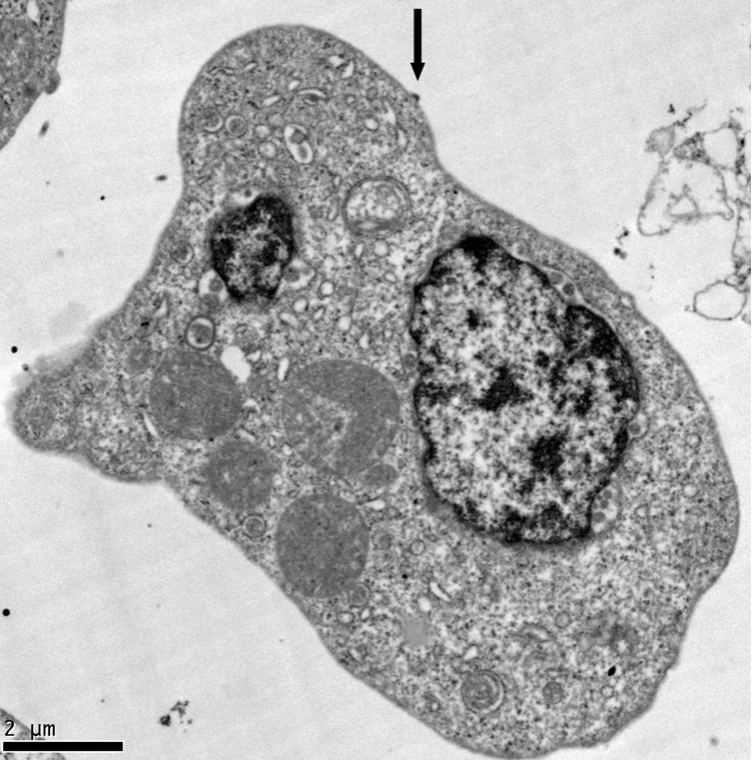
**TEM of triply-infected K7 cell**. HIV-1 particle budding from the plasma membrane (arrow).

**Figure 7:**
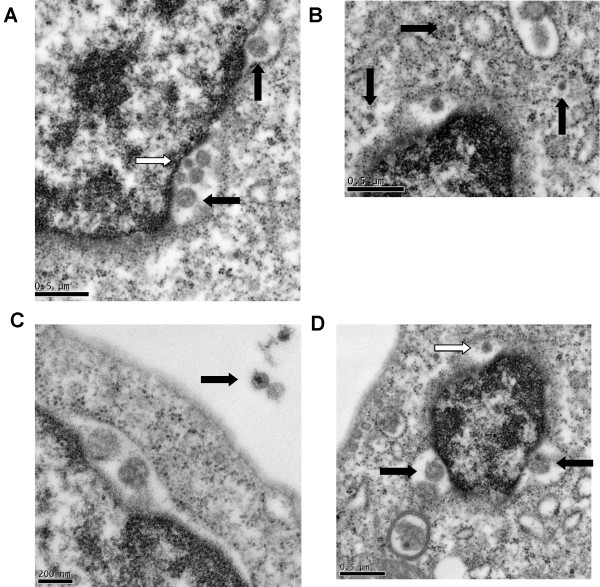
**Enlargements of triply-infected cell from Figure 6**. **A. **HHV-6 budding from the nuclear membrane (black arrows) and HCV in the perinuclear space (white arrow). **B. **HCV particles in the cytoplasm. **C. **HIV-1 particle outside the cell. **D. **HHV-6 (black arrows) and HCV (white arrow) in the perinuclear space.

As noted above, the K7 cell culture contained all three viruses. HIV-1 and HCV were seen outside the cells (Figure 5), and the cells contained both complete and incomplete HCV virions. One example of a triply infected cell is shown in Figure 6. Incomplete HHV-6A particles were seen budding from the nucleus (Figures 7A–7D). HIV-1 was seen budding from the plasma membrane of the cell (Figure 6). In addition, HCV virions were seen in the cytoplasm and in the vicinity of budding HHV-6A particles (Figure 7B), and other incomplete HCV particles were also seen in the perinuclear space (Figures 7A and 7D). These HCV particles were approximately 70 to 100 nm in size. HIV-1 was present outside but adjacent to or in the vicinity of the infected cell (Figure 7C).

## Discussion

Studies that have compared HCV-infected patients with HCV-HIV-1 infected patients have observed that there is a longer half-life of HCV, and up to ten times higher HCV RNA levels in serum or liver [2,4]. Co-infected patients are more likely to develop cirrhosis, which may rapidly progress to acute disease [14]. The mechanisms for these effects are unclear, but loss of immune function by HIV-1 infection is thought to be a significant contributing factor. However, most studies of HAART suggest that it does not significantly affect the levels of HCV RNA [2], meaning that HIV-1 may have little effect on HCV RNA production. These studies are confusing, since evidence for HCV replication is absent, and HCV RNA levels do not correlate with the levels of infectious HCV in the blood. Unfortunately there is no commonly used system to determine these levels.

Other studies that have looked at the effects of HCV on HIV-1 infected individuals have also shown conflicting results. Early studies suggest that HCV increases the rate of progression to AIDS in co-infected individuals, but others have not seen this [2,3]. A large Swiss study showed HCV serum-positivity was associated with more likely progression to AIDS and a less likely increase of CD4+ cell count after therapy [15,16]. Similar determinations can be made by comparing other viral infections such as HHV-6 and HIV. A strong case can be made for HHV-6 as a co-factor in the development of AIDS. Other short-term studies have not found a correlation between CD4+ cell number and negative consequences due to HCV infection [17,18].

*In vitro *studies of HIV-HCV co-infected cells have been hampered by a lack of a system that can replicate both viruses. Laskus *et al*. [19] showed that HIV-1 stimulates HCV production in macrophages *in vitro*. The system, however, did not support HCV replication for extended periods of time. Macrophages, although they stay alive for extended periods of time, do not synthesize DNA and replicate.

Initial *in vitro *studies of HIV-HHV co-infected T-cells showed that HHV-6 increased HIV-1 production and cell death [20]. Later studies showed that HHV-6 could decrease HIV-1 production in some cell types, such as dendritic cells [21] and macrophages [22]. This difference in virus production may be accounted for by differences in activities of the LTR of HIV-1.

Patients and clinically normal individuals are frequently infected with multiple viruses. It is therefore important to understand the implications of simultaneous infection by multiple viruses. Since co-infections with HIV, HCV, and HHV-6 are frequently seen in the same individual, the development of a system to study the effects of the interactions among these viruses at the level of a single cell is important. Our experience in human viruses allowed us to develop this system. We realize that the occurrence of triple infections of single cells may be a rare event, yet it also establishes the fact that immunity and dominance are also limited statistical phenomena.

Our system for growing HCV starts with the infection of macrophages obtained from human cord blood. Macrophages are distributed all over the body, and they perform specialized functions, e.g., Kupffer's cells of liver, dendritic cells of skin, astrocytes and microglial cells of the nervous system. In addition, the infection of our human neuronal precursor cells with CIMM-HCV may be similar to the infection of macrophages with HIV-1. These macrophages may become multiply infected and function as a reservoir. Other investigators have shown the presence of HCV in human brains in the post-mortem analysis [23,24]. Similarly, HIV-1 has also been reported in the brain environment [25-27]. These observations may partly explain the diminished cognitive function, depression and fatigue in these individuals. The presence of HIV-1 and HCV in the same cell may greatly aggravate the malady.

Our TEM analysis showed HHV-6A and HCV in the same cells, with extracellular HIV-1 particles budding or adjacent to the infected cells. Since HIV-1 assembly is completed at the plasma membrane level, their presence is only seen outside the cells. The assembly and synthesis of HHV-6A begins in the nucleus, the virion acquires a membrane as it passes through the nuclear membrane, and it matures in the cytoplasm. The synthesis and maturation of HCV is presumed to occur in the cytoplasm. This, in our opinion, is an evolving concept. We have seen HCV particles in the nucleus, in the perinuclear space, in the cytoplasm, in vesicles, and outside cells. The presence of HCV particles in the perinuclear space may indicate that at least partial synthesis of this virus may occur in the nucleus. Recent suggestions that some nuclear proteins bind to HCV RNA support this possibility [28]. The TEM results presented in Figures 6 and 7 suggest that HCV and HHV-6A may have similar or identical sites of replication. Others have proposed that HCV synthesis may occur in the perinuclear space [29,30], which is unlikely.

Another question addressed by these experiments was to conclusively demonstrate that HCV can replicate well in T-cells. There was a difference in the level of virus replication when compared to B-cells but, as shown in the electron micrographs, it replicates well in T-cells. HCV has been shown to infect T-cells in addition to other cell types in infected individuals [31], so it is not surprising that significant production of HCV particles occurs *in vitro*. The claims of levels of virus titers for many human viruses are merely claims. There are no definitive and reproducible methods for titrating biologically active HIV-1, HTLV-I, HTLV-II, HHV-6, or HCV with accuracy. We generally use the RNA levels as a relative indicator of virus production.

This study, in addition to our previous reports, supports the notion that HCV infects multiple hematopoietic cell types, *viz *monocytes-macrophages, B-cells, and T-cells. We have also reported that neuronal cells, endothelial cells (hepatocytes), and Kupffer's cells of liver can also be infected. Replication of HCV in liver cells is generally very low, which may be clinically relevant.

Since HCV, HIV-1, and HHV-6A can coexist in culture, none of these viruses prevent the others from infection and replication. However, these effects may be variable due to a number of factors that affect replication. This has relevance to viral protein production that may induce or produce pathology. How the viruses interact will be the subject of later work.

## Methods

### Cell culture and viruses

HIV-1 [9,11], HHV-6A [10,32], and HCV [5] are routinely isolated in our laboratories at the California Institute of Molecular Medicine (CIMM), Ventura, CA. Freshly isolated viruses were used for this study. Stocks of these isolates were stored in aliquots of 1 ml at -70°C for experimental use. CEM cells were previously obtained from the American Type Culture Collection (ATCC), Bethesda, MD.

Transmission experiments were carried out using our standard protocol. Briefly, CEM cells were seeded at 10^5 ^cells/5 ml in complete medium supplemented with 10% fetal bovine serum containing 5 ng of polybrene, and incubated overnight. To infect these cells, they were centrifuged, the media was discarded, and the cells were re-suspended in 1 ml of stock virus, incubated at 37°C in a 5% CO_2 _atmosphere. Cell cultures were centrifuged, and the culture supernatants were filtered through 0.45 μ filter membranes for assays.

The CEM cells were infected sequentially using HCV followed by HHV-6A and HIV-1 (Figure 1). Cell cultures were incubated overnight each time post infection. Aliquots of all the infected cells and cell culture supernatants were saved in liquid nitrogen and at -70°C, respectively.

### Detection of viruses using RT-PCR and PCR analyses

RNA was purified from the cell culture supernatants and nested RT-PCR was performed to amplify HCV RNA [5] or HIV-1 RNA [33]. To detect HHV-6A, DNA was purified from the cell culture supernatants and PCR was performed using the U16-17F and U16-17R primers [34]. The primers used for the PCR and RT-PCR experiments are listed in Table 2.

### Transmission electron microscopy (TEM)

Cultured cells were placed in fixative provided by the electron microscopy group at the City of Hope, Duarte, CA. The fixed samples were shipped to them overnight for sectioning and analysis.

The cells analyzed by TEM were well fixed, although cut thick they were easy to interpret. Since HIV-1 particles are assembled at the plasma membrane level they are only found either budding from the cell membrane or outside the cells. Hence, they do not appear inside the triply infected cells. The intracellular presence of HHV-6A and HCV is impressive.

## Competing interests

All intellectual rights are reserved by the California Institute of Molecular Medicine (CIMM), and all aspects of this work were performed by CIMM. There are no competing interests between California Lutheran University or any other body and CIMM.

## Authors' contributions

K.A.S., A.G., and R.G. performed biological work. J.G.P. and A.S.K. performed the clinical work, recruitment of patients, and procurement of specimens. K.A.S. and A.G. performed molecular work. S.Z.S. and D.R. designed and conducted experiments, analyzed the data, and wrote the manuscript. All of the authors have read and approved the final manuscript.
